# Retraction Note: Musical practice and BDNF plasma levels as a potential marker of synaptic plasticity: an instrument of rehabilitative processes

**DOI:** 10.1007/s10072-021-05283-2

**Published:** 2021-05-04

**Authors:** Alessandro Minutillo, Gabriele Panza, Massimo Carlo Mauri

**Affiliations:** 1 Department of Neurosciences and Mental Health, Fondazione IRCCS Ca’ Granda Ospedale Maggiore Policlinico, Milan, Italy; 2 Department of Pathophysiology and Transplantation, University of Milan, Milan, Italy

**Retraction Note: Neurological Sciences (2021) 42:1861–1867**

10.1007/s10072-020-04715-9

The authors retracted this article, taken from the dissertation (graduation thesis) of co-author Alessandro Minutillo, as they had not been authorized to publish the data. The Department of Brain and Behavioural Sciences of the University of Pavia have confirmed that they are the rightful owner of the data as reported in this article. All authors agree with this retraction. 

